# Reference values for body composition and physical fitness of young Brazilian elite soccer players

**DOI:** 10.3389/fphys.2023.1285952

**Published:** 2023-11-02

**Authors:** Daniel Leite Portella, Ruben Vidal-Espinoza, Jose Sulla-Torres, Luis Felipe Castelli Correia de Campos, Rossana Gomez-Campos, Marco Cossio-Bolaños

**Affiliations:** ^1^ School of Physical Education, Master Program in Innovation in Health Education, Universidade Municipal de São Caetano do Sul, São Caetano do Sul, Brazil; ^2^ Escuela de Ingenieria de Sistemas, Universidad Católica Silva Henríquez, Santiago, Chile; ^3^ Universidad Católica de Santa María, Arequipa, Peru; ^4^ Departamento de Ciencias de la Educación, Universidad del Bio Bio, Chillán, Chile; ^5^ Carrera de Ciencias de la Actividad Física, Facultad de Salud, Universidad San Ignacio de Loyola, Lima, Peru

**Keywords:** body composition, physical fitness, young, soccer player, percentile, Brazil

## Abstract

**Objective:** a) to verify whether body composition and physical fitness should be analyzed by chronological age and/or maturity stage in young Brazilian soccer players and b) to propose reference values for the evaluation of body composition and physical fitness by maturity stage in elite soccer players.

**Methods:** A descriptive-correlational study was carried out in 206 young Brazilian soccer players (11–16 years old). The sample selection was non-probabilistic by convenience. Weight and standing height were evaluated. Body composition (BC) was assessed by dual X-ray absorptiometry (DXA). BC indicators [(percent fat (%F), Fat-free mass (FFM), Fat mass (FM) and Bone mass (BM)] were extracted. For physical fitness (PF), we applied the Flexibility (cm) sit and reach tests, explosive strength tests [Counter Movement Jump CMJ (cm) and horizontal jump HJ (cm)], speed [Speed 10, 20, 30 and 40 m (seconds)] and Yo-Yo endurance level I test. Percentiles were constructed for BC and PF using the LMS method [L (Lambda; skewness), M (Mu; median) and S (Sigma; coefficient of variation)].

**Results:** The explanatory power between chronological age (CA) with BC was: FM (R2 = 0.03%), FFM (*R* ^2^ = 0.66%) and BM (*R* ^2^ = 0.62%), while between maturity status (MS) with BC were: FM (*R* ^2^ = 0.04%), FFM (*R* ^2^ = 0.71%) and BM (*R* ^2^ = 0.66). The explanatory power between the CA with the physical fitness tests ranged from: (*R* ^2^ = 0.22–0.62%). While between MS with physical fitness the values ranged from: (*R* ^2^ = 0.23–0.64%). Percentiles per MS (P3, P5, P10, P15, P25, P50, P75, P85, P90, P95 and P97) were proposed for both BC and PF.

**Conclusion:** The results of the study have shown that the evaluation of BC and PF of young soccer players should be performed by controlling for MS rather than for CA. The inclusion of a non-invasive method to control MS by means of percentiles during puberty may contribute to the development of retention and exclusion of young soccer players, thus, they may have a better chance of achieving sporting success.

## 1 Introduction

In recent years, the assessment and monitoring of body composition (BC) and physical fitness (PF) in young soccer players has gained a wide space in sport sciences. For example, BC in athletes can help optimize competitive performance and monitor the success of training and nutrition regimens (Rodriguez et al., 2009; Suarez-Arrones et al., 2019). Furthermore, body compartments such as fat-free mass (FFM) strongly contribute to strength and power performance (Deprez et al., 2015). While contrarily, a high fat percentage (%F) impairs sport excellence in athletes (Gil et al., 2007).

On the other hand, the evaluation of PF is fundamental to be able to apply the results to individual sports training planning (Salinero et al., 2013). It can even serve as an effective tool to improve feedback with players, as well as to monitor progress during the stages of sports training (Romero-Caballero et al., 2021).

In general, several studies have highlighted the use of anthropometric measurements, BC, assessment of physiological parameters, PF and motor competence in young soccer players as indicators in talent detection (Stølen et al., 2005; Lidor et al., 2009; Coelho et al., 2010; Williams et al., 2014; Hohmann and Siener, 2021). To this end, reference data from various PF and BC tests are needed to assist coaches and athletes in the identification, selection and development of athletic talent (Lloyd and Oliver, 2012).

In that context, percentiles are a fundamental tool, often in everyday use in sports science. This is useful for professionals working in various sport modalities, especially for the evaluation and monitoring of athletes in clinical, and field settings (Santos et al., 2014). Its importance lies in the use of percentile values as approximate benchmarks for talent identification and development (Lesinski et al., 2020), as well as it can serve coaches to effectively adjust training interventions to players’ strengths and weaknesses (Paul and Nassis, 2015).

Indeed, to the best of our knowledge, there are multiple studies in school children and adolescents describing anthropometric, PF and BC percentiles according to age and sex (Hobold et al., 2017; Tomkinson et al., 2018; Iglesias-Soler et al., 2021; Ma et al., 2022). However, these datasets cannot be used with young athletes because, by definition, talented youth are equal to or above the 90th percentile of the respective general population (Williams et al., 2014). Therefore, it is essential to develop reference values for athletes and especially for young soccer players in the process of growth and development.

To our knowledge, few studies have been identified that propose referential values in young soccer players. However, these studies do not cover a wide age range, usually, they have been limited to some physical tests (Lesinski et al., 2020; Selmi et al., 2020; Romero-Caballero et al., 2021; Smpokos et al., 2022), or have been proposed to separately analyze physical growth and BC (Carrasco-López et al., 2018; Carrasco-López et al., 2021), which limits their use and application.

Furthermore, based on the fact that, during adolescence, soccer players present significant individual differences in BC and physical performance (Nikolaidis and Karydis, 2011), studies in general have pointed out that for talent identification and for the development of training in sports, maturity status (MS) control should be considered before CA (Lloyd et al., 2015; Gómez-Campos et al., 2019; Selmi et al., 2020). Therefore, this study hypothesizes that the assessment of BC and PF of young soccer players should be analyzed by MS and not by Chronological age, since interindividual variations of these indicators during biological maturation are significant. Therefore, it is recommended that professionals working in youth soccer measure players’ MS estimates every 3–4 months during an annual season, with special attention on players approaching peak height velocity (PHV) and during PHV (Towlson et al., 2021).

For example, speed and flexibility suggest peak gains before PHV in boys (Beunen et al., 1988), while in strength and power tests reached peak gains after PHV (Malina et al., 2004), however, peak velocity at peak aerobic capacity (VO_2_max) occurs at PHV in both sexes (Slough et al., 2013).

Therefore, this study aimed to a) verify whether BC and PF should be analyzed by CA and/or MS in young Brazilian soccer players and b) propose reference values for the evaluation of BC and PF by MS in elite soccer players.

## 2 Materials and Methods

### 2.1 Type of study and sample

A descriptive-correlational study was carried out in 206 young Brazilian soccer players (11–16 years old). The sample selection was non-probabilistic by convenience. The young soccer players belonged to the lower categories of a professional club in Sao Paulo (Brazil). These young players trained five times a week (1 competition day and one rest day). The training sessions were about 90 min per day. At the time of the study, all players were competing at the national level in the Brazilian soccer confederation.

All young soccer players were healthy and did not suffer from any type of illness or physical injury that could affect the anthropometric assessments, DXA scan and physical test results.

The study was conducted in accordance with the guidelines established in the Helsinki declaration for human subjects. The study was approved by the Ethics and Research Committee of the Municipal University of São Caetano do Sul, São Paulo (Brazil), with protocol number 41232214.2.0000.5510.

Players who were registered in the club with a minimum of 2 years of experience in the grassroots categories were included. Players who presented any type of physical injury that affected their performance in the anthropometric and physical measurements were excluded. As well as those young people who were 17 years of age or older and had been considered for the professional team.

### 2.2 Techniques and procedures

The anthropometric measurements, DXA scan and PF assessment were performed at the Club’s facilities in April 2016. All evaluations were performed in three stages: stage 1: anthropometric evaluation and DXA scanning, stage 2: physical test evaluation (flexibility, speed, horizontal jump SH, vertical jump with counter movement CMJ) and stage 3: evaluation of the Yo-Yo test Endurance level 1.

**Stage 1:** Anthropometric measurements for body weight and standing height were measured according to the standard procedures described by Ross and Marfell-Jones (1991). All variables were measured in the Club’s facilities. Athletes were barefoot and dressed in athletic clothing. Body weight was assessed using a portable electronic scale (Tanita Inner Scan BC 532, Tokyo, Japan) with an accuracy close to 0.1 kg. Standing height was assessed according to the Frankfurt plane, a portable stadiometer (Seca 213, Hamburg, Germany) with an accuracy of 0.1 cm was used. To verify the reliability of these anthropometric measurements, all the young people were evaluated twice. The technical measurement error (TME) showed values below 1%.

The BC scan was performed by means of DXA. The equipment used was Hologic Discovery model (Hologic 4500-A Hologic Inc., Bedford, MA, United States). The equipment was calibrated according to the recommendations of the international densitometry society (Schousboe et al., 2013). Hologic APEX software with Windows XP operating system was used on all three devices (version 13.3.01) for data analysis. Headless whole-body mode was selected to scan the athletes.

Before performing the scan, all athletes were given a brief description of the procedure and were prohibited from wearing jewelry and the presence of some types of metal on the body, which should be removed before the scan. To start the body scan, the athletes were instructed to remain in the supine position with arms extended at the sides of the body and with the knees and ankles fastened with a Velcro strap (to secure the predetermined position). One of the evaluators aligned the landmarks according to the lines displayed by the software. Body composition indicators [(percent fat (%F), Fat-free mass (FFM), Fat mass (FM) and Bone mass (BM)] were extracted. To ensure DXA scanning, 10% of the total sample (21 young people) was evaluated twice. The TME was 1.5%.

**Stage 2:** CF was evaluated at the club’s facilities. All athetes performed a 10–15 min warm-up (jogging exercises with changes of pace, and flexibility). The order of the tests was: Flexibility, Horizontal jump (HJ), Counter movement jump (CMJ), and speed (10, 20, 30 and 40 m).

The modified sit and reach test: Evaluates the flexibility of the dorso-lumbar region in (cm). A wooden Wells bench with a scale of 0–50 cm was used, following the recommendations of (Hoeger and Hopkins, 1992). Three attempts were made, recording the best value. The TME was 0.8%.

Explosive strength was evaluated through two tests: HJ and CMJ. The HJ (cm) was evaluated following the recommendations of Castro-Piñero et al. (2010). A 3-m metric tape measure with an accuracy of 0.1 cm was used. Athletes were positioned feet together, keeping their feet shoulder width apart. Then, with feet together, the athlete performed a forward motion with as much momentum as possible to get as far as possible from the starting line. Athletes made three attempts. The best result was recorded. The CMJ jump (cm) was performed according to the recommendations of Bosco (1999). A branded jumping platform (Cefise, Bipodal force plate VJB, Brazil) was used. Athletes were positioned on the platform with both feet on a contact grid with their hands on their hips. Participants were asked to jump as high as possible using a countermovement jump, keeping their hands on their hips and without bending their knees while in the air. The height of the jump was determined using Vertical Jump Power software, Cefise, Brazil. This test was measured twice. The TME was 1%.

The speed tests were evaluated by a 40 m straight line sprint with split times of 10, 20, 30 and 40 m. Players started the test from a standing position, keeping one foot forward (0.5 m behind the timing line). A photoelectric cell (Cefise, Speed Test Fit, Brazil) with four pairs of cells was used. Each tripod was located 0.75 m above the ground and placed 3 m in front of each other on both sides of the starting line (Pardos-Mainer et al., 2020). They were instructed to run as fast as possible, being encouraged at all times. Two attempts were made, 3 min of recovery was given between each attempt and the best time was recorded as the final result (Mainer-Pardos et al., 2021). The TME was 0.7%.

**Stage 3:** Yo-Yo Endurance test Level 1 (Yo-Yo E1): This is a progressive and maximal test that evaluates aerobic endurance. It was evaluated following the suggestions of Bangsbo (1994). It was evaluated on a soccer field with natural grass. Participants run in a back and forth direction on a 20-m out and 20-m return course. The initial speed for the level 1 endurance test is 8.0 km/h and increases by 0.5 km/h approximately every minute. An audio was used to guide the pace run based on sound signals (beep). The total distance covered by each athlete was considered as the final result. This test was evaluated only once.

Body mass index (BMI) was calculated using the formula: BMI = weight (kg)/height^2^ (m). Maturity status (MS) was calculated by means of the anthropometric technique proposed by Moore et al. (2015). This technique uses chronological age and standing height. The equation we used was: Males: maturity status (years) = −7.999994 + (0.0036124 × (age × height)).

### 2.3 Statistics

The normal distribution of the data was verified by the Kolmogorov-Smirnov test. The descriptive statistics of arithmetic mean (X), standard deviation (SD) and confidence interval (CI) were calculated. The relationships between variables were obtained using Pearson’s correlation coefficient. The explanatory power *R* ^2^ and standard error of estimation (SEE) were also calculated. Differences between ages and MS were verified by means of one-way Anova and Tukey’s test of specificity. In all cases a probability of *p* < 0.05 was adopted. Statistical analysis was performed in SPSS v.23.0. Percentile curves were created for the BC and for the PF tests by MS (P3, P5, P10, P15, P25, P50, P75, P85, P90, P95 and P97) using the LMS method [L (Lambda; skewness), M (Mu; median) and S (Sigma; coefficient of variation)] proposed by Cole et al. (2000). The LMS Chart Maker version 2.3 software (Pan and Cole, 2006) was used to plot the curves.

## 3 Results

The anthropometric characteristics aligned by CA and MS are shown in Table 1. In the comparisons by CA, differences in weight and BMI begin to appear at 14 years of age. However, in height and % body fat, differences appear 1 year earlier (at 13 years of age). Regarding MS, significant differences were observed in weight, height, BMI and % fat in the four maturation stages (*p* < 0.05). In general, a significant increase in weight, height and BMI was observed as CA and MS increases. While the opposite happens in %F, where these values decrease as CA and MS increase.

**TABLE 1:** Anthropometric and % body fat characteristics aligned by chronological age and maturity status.

Indicator		Weight (kg)			Height (cm)			BMI (kg/m^2^)			Percentage of fat (%F) DXA			
	n	X	SD	CI	X	SD	CI	X	SD	CI	X		SD	CI
CA (years)														
11	25	39.9	5.3	37.8–42.1	147.5	5.3	145.3–149.7	18.3	1.7	17.6–19.0	19.3		5	17.2–21.3
12	32	41.6	6.3	39.3–43.9	150.4	5.9	148.2–152.5	18.3	1.9	17.7–19.0	18		8.7	14.9–21.2
13	29	46.2	6.1	43.9–48.6	156.8^ab^	7.5	153.9–159.6	18.8	1.8	18.1–19.4	14.4^a^		5.9	12.2–16.7
14	44	58.3^abc^	8.3	55.8–60.8	166.7^abc^	5.3	165.1–168.3	20.9^abc^	2.2	20.3–21.6	11.8^ab^		3.3	10.8–12.8
15	45	65.7^abcd^	9.9	62.7–68.6	172.1^abcd^	5.2	170.5–173.6	22.1^abc^	2.4	21.4–22.8	10.4^abc^		2.2	9.8–11.1
16	31	68.7^abcd^	6.1	66.4–70.9	177.1^abcde^	4.5	175.4–0.178.7	21.9^abc^	1.7	21.3–22.5	9.6^abc^		1.8	8.9–10.2
Maturity status (APHV)														
−1	50	40.1	4.6	38.7–41.5	149.1	4.2	147.8–150.4	18	1.6	17.5–18.5	20.1		6.9	18.0–22.2
0	78	52.1^†^	7.8	50.3–53.8	161.4^†^	7.2	159.8–163.0	19.9^†^	2	19.4–20.4	13.0^†^		4.9	11.9–14.1
1	43	65.8^†.††^	9.2	63.0–68.6	172.2^†.††^	4.2	170.9–173.5	22.1^†.††^	2.4	21.4–22.8	10.2^†.††^		2.2	9.5–10.9
2	35	70.1^†.††^	7	67.6–72.6	176.9^†.††.†††^	3.4	175.6–178.1	22.4^†.††^	1.9	21.7–23.1	9.7^†.††^		1.7	9.1–10.3

X: mean, SD: standard deviation, CI: confidence interval, BMI: body mass index, DXA: dual X-ray absorptiometry, CA: chronological age, MS: maturity status, a: significant difference relative to 11 years, b: significant difference relative to 12 years, c: significant difference relative to 13 years, d: significant difference relative to 14 years, e: significant difference relative to 15 years, †: significant difference relative to -1APHV, †††: significant difference relative to 0APHV, †††††: significant difference relative to +1APHV.

The linear regression values of BC and PF of young soccer players alienated by CA and MS are shown in Table 2. In BC, it is observed that FM was higher by 1% when alienated by MS relative to CA, while the power of explanation by MS increased by 4% and 5% (BMC and FFM) *versus* CA. As for PF, the *R* ^2^ evidenced higher values from 1% to 2% for MS relative to CA. In general, both BC and PF presented better results in terms of explanation by MS and lower EES.

**TABLE 2:** Simple linear regression values between chronological age and maturity status.

Variables	Chronological age (years)			t	*p*	Maturity status (APHV)			t	*p*
	R	*R* ^2^	SEE			R	*R* ^2^	SEE		
Body composition										
FM (kg)	0.18	0.03	1.52	51.98	0.000	0.19	0.04	1.44	3.89	0.000
FFM (kg)	0.81	0.66	0.91	35.59	0.000	0.84	0.71	0.79	-19.75	0.000
BM (kg)	0.79	0.62	0.96	44.77	0.000	0.82	0.66	0.85	-17.29	0.000
Physical Fitness										
Flexibility (cm)	0.58	0.34	1.26	25.6	0.000	0.59	0.35	1.19	-9.11	0.000
CMJ (cm)	0.79	0.62	0.95	16.45	0.000	0.8	0.64	0.88	-17.87	0.000
HJ (cm)	0.78	0.61	0.97	8.29	0.000	0.79	0.62	0.91	-17.2	0.000
Speed 10 m (sec)	0.75	0.56	1.03	27.77	0.000	0.76	0.57	0.96	16.9	0.000
Speed 20 m (sec)	0.65	0.42	1.18	27.81	0.000	0.66	0.44	1.1	12.95	0.000
Speed 30 m (sec)	0.68	0.46	1.14	25.01	0.000	0.69	0.48	1.06	14.03	0.000
Speed 40 m (sec)	0.73	0.53	1.06	29.09	0.000	0.74	0.55	0.99	16.08	0.000
Yo-Yo Endurance (m)	0.47	0.22	1.36	19.74	0.000	0.48	0.23	1.28	-6.92	0.000

SEE, standard error of estimation; FM, fat mass; FFM, Fat-free mass; BM, bone mass; CMJ, countermovement jump; HJ, horizontal jump.

**FIGURE 1:**
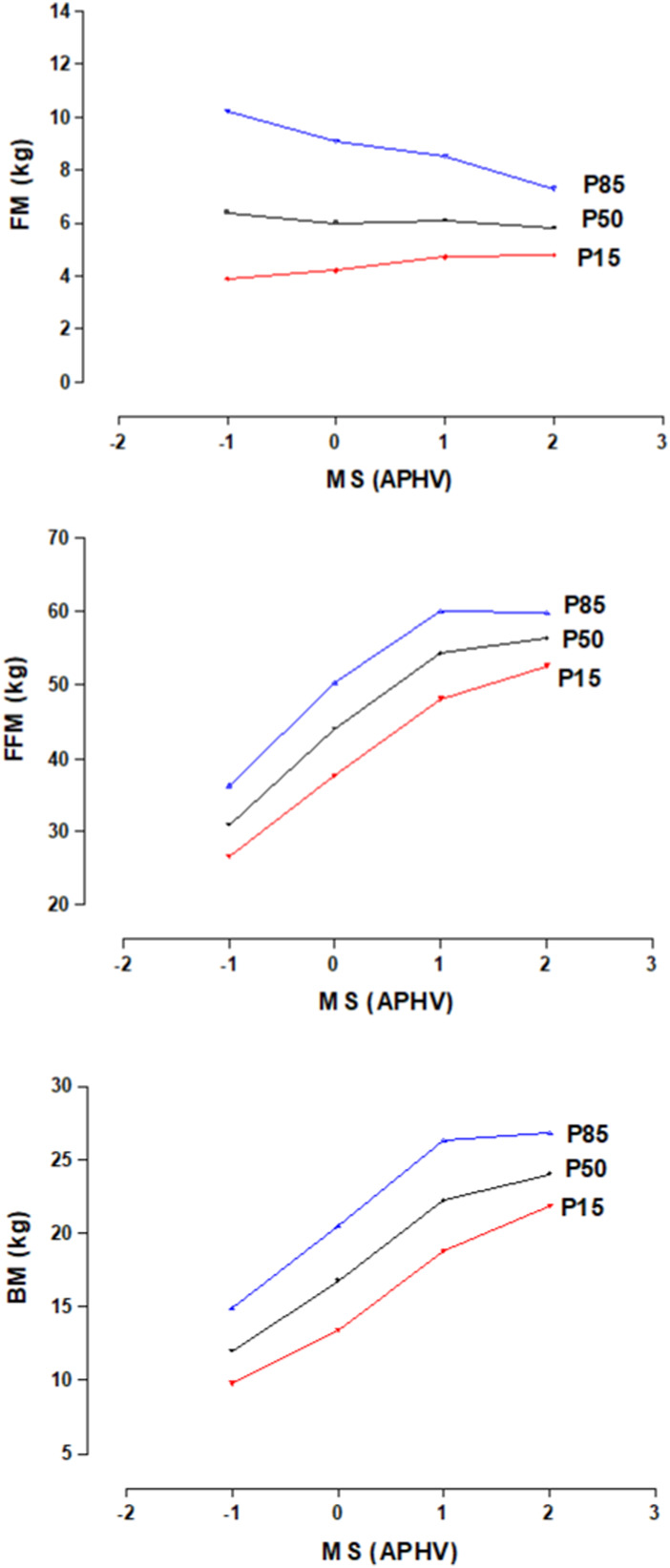
Body composition percentile graphs of young soccer players by maturity stage.

The distribution of percentiles (P3, P5, P10, P15, P25, P50, P75, P85, P90, P95 and P97) by MS for BC is shown in Table 3 and for PF in Table 4. These values are categorized from -1APHV, 0PHV, 1PHV and 2PHV. The median values for FM reflect relatively stable values according to maturity status categories (-1APHV, 0PHV, 1APHV and 2APHV), whereas in FFM and BM the values are ascending as MS increases. The percentile plots of the BC are seen in Figure 1.

**TABLE 3:** Percentiles for body composition (DXA) of young soccer players according to maturity status.

MS (APHV)	L	M	S	P3	P5	P10	P15	P25	P50	P75	P85	P90	P95	P97
	FM (kg)													
−1	0.04	6.37	0.46	2.7	3.0	3.5	3.9	4.7	6.4	8.7	10.2	11.4	13.4	14.8
0	−0.48	5.97	0.37	3.3	3.5	3.9	4.2	4.7	6.0	7.8	9.1	10.2	12.3	14.0
1	−0.84	6.09	0.28	3.9	4.1	4.5	4.7	5.1	6.1	7.5	8.5	9.3	10.9	12.1
2	−0.99	5.8	0.19	4.3	4.4	4.7	4.8	5.1	5.8	6.7	7.3	7.7	8.5	9.1
	FFM (kg)													
−1	−0.65	30.86	0.15	23.8	24.6	25.8	26.6	28	30.9	34.3	36.3	37.9	40.3	42.1
0	1.33	44.04	0.14	32.1	33.7	36.0	37.6	39.9	44.0	48.1	50.2	51.6	53.7	55.0
1	1.64	54.31	0.11	42.6	44.2	46.5	48.1	50.3	54.3	58.1	60.1	61.4	63.4	64.6
2	1.18	56.25	0.06	49.5	50.4	51.7	52.6	53.9	56.3	58.6	59.9	60.8	62.1	62.9
	BM (kg)													
−1	−0.37	11.98	-0.37	8.4	8.8	9.4	9.8	10.5	12.0	13.8	14.9	15.7	17.0	18.0
0	0.57	16.78	0.57	10.9	11.6	12.7	13.4	14.6	16.8	19.1	20.5	21.4	22.8	23.7
1	−0.03	22.23	-0.03	16.4	17.1	18.1	18.8	19.9	22.2	24.8	26.3	27.4	29.0	30.2
2	−1.27	24	-1.27	20.3	20.7	21.3	21.8	22.5	24.0	25.7	26.8	27.5	28.8	29.7

P: percentile, L: Lambda, M: median, S: Sigma, FM: fat mass, FFM: fat-free mass, BM: bone mass.

**TABLE 4:** Percentiles for physical fitness tests of young soccer players according to maturity status.

MS (APHV)	L	M	S	P3	P5	P10	P15	P25	P50	P75	P85	P90	P95	P97
	Flexibility (cm)													
−1	0.10	25.13	0.18	17.9	18.7	20	20.9	22.3	25.1	28.3	30.1	31.4	33.5	34.9
0	0.82	30.2	0.19	19.6	20.9	22.9	24.2	26.3	30.2	34.2	36.4	37.9	40.1	41.6
1	1.54	34.38	0.18	21.4	23.2	25.9	27.6	30.1	34.4	38.4	40.5	41.9	43.9	45.1
2	2.20	36.05	0.13	25.1	26.8	29.1	30.6	32.6	36.1	39.1	40.6	41.6	43.1	44
	CMJ (cm)													
−1	−0.23	31.74	0.13	25	25.7	26.9	27.8	29.1	31.7	34.7	36.4	37.7	39.6	40.9
0	−0.37	36.86	0.11	30	30.7	31.9	32.8	34.2	36.9	39.9	41.6	42.9	44.8	46.1
1	−1.1	41.37	0.09	35.3	35.9	37	37.8	39	41.4	44.1	45.8	47	48.9	50.2
2	−2.82	45.12	0.07	40.2	40.7	41.5	42.1	43.1	45.1	47.6	49.2	50.3	52.3	53.7
	HJ (cm)													
−1	1.19	181.29	0.1	144.9	149.6	156.7	161.4	168.4	181.3	194	200.8	205.3	212	216.3
0	1.98	207.02	0.09	168.9	174.1	181.9	187	194.2	207	219.1	225.3	229.5	235.4	239.2
1	1.1	224.19	0.07	193.5	197.4	203.4	207.4	213.3	224.2	235.1	240.9	244.8	250.7	254.4
2	−0.65	231.54	0.05	209.7	212.2	216.3	219.1	223.3	231.5	240.3	245.2	248.7	253.9	257.4
	Speed 10m (seconds)													
−1	0.02	2.08	0.05	1.87	1.9	1.94	1.97	2.01	2.08	2.15	2.18	2.2	2.24	2.26
0	0.02	1.97	0.04	1.8	1.83	1.86	1.88	1.91	1.97	2.02	2.05	2.07	2.1	2.12
1	0.03	1.91	0.03	1.79	1.8	1.83	1.84	1.87	1.91	1.95	1.97	1.99	2.01	2.02
2	0.03	1.84	0.03	1.74	1.75	1.77	1.78	1.8	1.84	1.87	1.89	1.9	1.92	1.93
	Speed 20 m (seconds)													
−1	−0.01	3.62	0.09	3.09	3.15	3.24	3.31	3.41	3.62	3.86	4	4.1	4.26	4.37
0	−0.03	3.44	0.07	3.09	3.12	3.18	3.23	3.29	3.44	3.61	3.72	3.8	3.94	4.05
1	−0.01	3.25	0.04	3.02	3.04	3.09	3.12	3.16	3.25	3.35	3.4	3.43	3.49	3.52
2	0.05	3.14	0.03	2.97	2.99	3.03	3.05	3.09	3.14	3.2	3.22	3.24	3.27	3.28
	Speed 30 m (seconds)													
−1	0.02	4.93	0.06	4.3	4.38	4.51	4.59	4.71	4.93	5.14	5.24	5.32	5.43	5.49
0	0.00	4.7	0.05	4.25	4.31	4.39	4.45	4.54	4.7	4.87	4.97	5.03	5.13	5.2
1	0.04	4.55	0.04	4.17	4.22	4.3	4.35	4.42	4.55	4.66	4.72	4.75	4.81	4.84
2	0.12	4.4	0.03	4.1	4.15	4.22	4.26	4.32	4.4	4.47	4.5	4.52	4.55	4.57
	Speed 40 m (seconds)													
−1	−0.03	6.44	0.06	5.84	5.91	6.01	6.08	6.2	6.44	6.72	6.9	7.03	7.24	7.39
0	−0.01	6.02	0.05	5.44	5.51	5.62	5.69	5.8	6.02	6.25	6.37	6.46	6.6	6.69
1	0.04	5.81	0.04	5.27	5.35	5.46	5.54	5.64	5.81	5.97	6.04	6.09	6.17	6.21
2	0.12	5.63	0.03	5.19	5.27	5.38	5.43	5.51	5.63	5.72	5.77	5.79	5.83	5.86
	Endurance (m)													
−1	0.04	1,601.29	0.19	1,107	1,160	1,246	1,307	1,404	1,601	1826	1958	2053	2,202	2,304
0	0.33	1804.72	0.18	1,273	1,333	1,429	1,496	1,599	1805	2027	2,153	2,242	2,378	2,469
1	−0.43	1978.91	0.15	1,529	1,577	1,655	1711	1798	1979	2,187	2,312	2,402	2,545	2,645
2	−2.01	1981.85	0.1	1,682	1712	1762	1799	1857	1982	2,137	2,236	2,312	2,441	2,537

P, percentile; L, lambda; M, median; S, sigma; CMJ, countermovement jump; HJ, horizontal jump.

**FIGURE 2:**
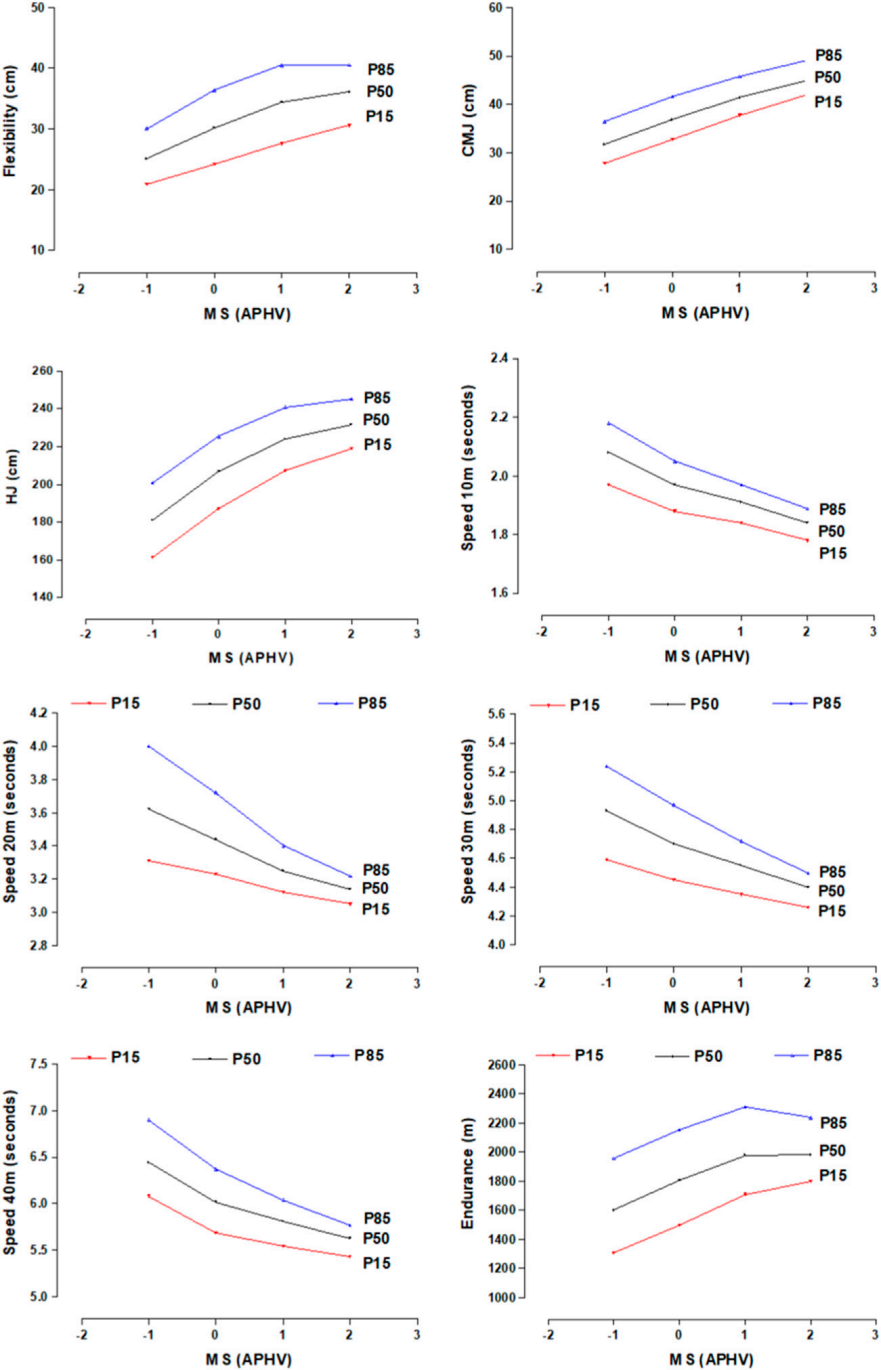
Percentile graphs of the physical fitness of young soccer players by maturity stage.

The PF test values reflect better physical performances as MS categories increase in the eight physical tests (flexibility, CMJ, HJ, speed 10, 20, 20, 30, 40 m and Endurance). The PF percentile plots of the eight tests are observed in Figure 2.

## 4 Discussion

The first objective of this study was to verify whether BC and PF should be analyzed by CA and/or MS in young Brazilian soccer players. The results of this study have shown that MS better explains BC and PF in young Brazilian soccer players.

These findings are similar to other studies that have investigated in non-sport school populations (Sherar et al., 2007; Kusari et al., 2023; Pezoa-Fuentes et al., 2023), sports (Müller et al., 2017; Gryko, 2021) and especially in young soccer players (Asadi et al., 2018; Müller et al., 2018; Peña-González et al., 2022; Ginés et al., 2023).

These evidences found in this study may be important for coaches, technicians, physicians, physiotherapists, and directors of youth soccer academies, as the analysis of BC and PF should be analyzed by MS and not by CA. In fact, differences between individuals in MS, are related to physical performance despite the expectations of coaches (Peña-González et al., 2022). For in general, participation in youth team sports is mainly based on CA categories that often encompass 2-year age groups, whose age ranges present considerable variations in size, function and motor skill (Figueiredo et al., 2009).

Indeed, several professional soccer clubs and league governing bodies (e.g., English Premier League) have invested in the long-term development of young soccer players (Balyi and Hamilton, 2004), as they often overlooked youth progress without considering the potential interactions between size, maturity, function, and motor skills. These programs evidently relied on research that accounts for the influence of biological maturity (Mirwald et al., 2002; Balyi and Hamilton, 2004; Ford et al., 2011).

Therefore, some authors such as Malina et al. (2007), highlight that variations in physical size and performance during biological maturity plays a relevant role in young soccer players, as younger players with a more advanced state of maturity may be unable to cope with older ones. While players with older age and MS may perceive that they play at a disadvantage with younger players. This consistent practice in soccer suggests the systematic exclusion of late-maturing children, which often increasingly favors average and early-maturing children (Cunha et al., 2017).

From this perspective, based on the results achieved in this study, the second objective was to develop reference values for the evaluation of BC and PF by MS in young elite Brazilian soccer players.

These percentiles will allow the classification of BC and PF of young soccer players according to maturation levels (APHV). These APHV varied in four levels (-1APHV, 0APHV, +1APHV and +2APHV).

The distribution of the percentiles of the three body compartments (FM, FFM and BM) evidenced increases in FFM and BM as MS increases. In the case of FM, it remains relatively stable at the four levels of APHV. While, in FP, the eight tests (flexibility, CMJ, HJ, speed 10, 20, 30, 40 m and Endurance level 1) reflected better physical performance as MS increases.

In general, the percentiles proposed in this study can be useful to identify young soccer players with high, moderate and low levels in BC (FM, FFM and BM) and PF. For this, it is necessary to categorize soccer players, for example, a recent study highlights that soccer players performing above the 80th percentile can be classified as having good fitness and below the 20th percentile should be selected and introduced in fitness promotion programs (Golle et al., 2015).

In fact, based on these previous premises and in the absence of a reference or criterion to categorize the BC and PF performance of young soccer players, this research makes available cut-off points in four categories: <p15 as very low, between p15 to p50 low, p50 to p85 moderate and >p85 as high fitness.

In this context, the percentiles developed in sports science, especially in young soccer players in recent years have focused on the assessment of BC (Carrasco-López et al., 2021), explosive strength (Petridis et al., 2019; Romero-Caballero et al., 2021), Sprints (Selmi et al., 2020), strength/power, flexibility and aerobic endurance (Smpokos et al., 2022), anthropometric profile, explosive strength, strength endurance and agility (Lesinski et al., 2020).

All these references in general serve to implement and monitor intervention programs, to produce reports and decisions in the selection of sport talents (Cumming et al., 2017), to identify performance deficiencies in athletes (Smpokos et al., 2022). As well as, for the monitoring and identification of physical performance capacity in young soccer players (Selmi et al., 2020) and to evaluate the effectiveness and progress during training programs (Petridis et al., 2019; Romero-Caballero et al., 2021).

In fact, the references proposed here encompass a large number of these tests, which allow jointly assessing three body compartments and eight physical tests in young Brazilian soccer players.

This developed tool is a valuable contribution to the performance of youth soccer, especially in Brazil. It has particular importance for professionals (coaches, physical trainers) working in educational (in schools), athletic (in talent identification) and health (in professional soccer academies) environments (Smpokos et al., 2022). Also, calculations can be obtained in real time using the following link: http://reidebihu.net/soccer_players_brazil.php.

In essence, this study increases knowledge on the assessment of BC and PF performance in young Brazilian soccer players. However, a broader and deeper research on the nutritional status, race and socioeconomic status of young soccer players is required. For it is highlighted that nutrition plays a key role in the maturation process of athletes (Bergeron et al., 2015), especially in physiological and hormonal changes (Malina et al., 2015). Although education, employment and economic income level are factors responsible for disparities in general health (Glymour et al., 2014) and consequently may affect the sports performance of young soccer players.

This research has some weaknesses, which have to do with the type of study, since we used a cross-sectional design, which makes it difficult to analyze the causal relationships between variables. We also used a non-probabilistic sample selection, which limits the generalization of the results to other sociocultural contexts and it is possible that the prediction of MS through a non-invasive technique presents some biases in the MS of the soccer players studied. Future research should consider these aspects indicated above, with which it is possible to reduce the biases obtained in this study. They should also open new research possibilities in young soccer players.

In fact, it is necessary to highlight that the study also presents great strengths. These have to do with the sample included, given that the young soccer players belong to an elite professional club in Brazil (FIFA Club World Cup champion). On the other hand, BC assessment was explored by means of gold standard DXA equipment and PF tests reflected low Technical measurement error (TEM) (<1.5%). Overall, this is one of the first studies performed in Brazil, whose results may serve not only for talent detection, but also as a baseline for future comparisons in secular trend studies.

## 5 Conclusion

The results of the study have shown that the evaluation of BC and PF of young soccer players should be performed by controlling for MS rather than by CA. These findings suggest that reference values should be developed by MS. Therefore, their use and application can serve to identify somatic maturation levels among athletes and to control and monitor club training and nutrition programs. The inclusion of a non-invasive method to control MS by means of percentiles during puberty may contribute to the development of retention and exclusion of young soccer players, thus, they may have a better chance of achieving sporting success.

## Data Availability

The raw data supporting the conclusions of this article will be made available by the authors, without undue reservation.
